# Anastomosis within and between networks of *Rhizophagus irregularis* is differentially influenced by fungicides

**DOI:** 10.1007/s00572-023-01103-x

**Published:** 2023-01-21

**Authors:** Victor Hugo Rodriguez-Morelos, Maryline Calonne-Salmon, Stéphane Declerck

**Affiliations:** Université Catholique de Louvain, Earth and Life Institute, Mycology, Croix du Sud 2, Box L7.05.06, 1348 Louvain-La-Neuve, Belgium

**Keywords:** Arbuscular mycorrhizal fungi, Mycelial networks, Fungicide, Anastomosis

## Abstract

Arbuscular mycorrhizal (AM) fungi play key roles in soil fertility of agroecosystems. They develop dense extraradical mycelial (ERM) networks via mechanisms such as hyphal anastomosis. These connections between hyphae can be affected by agricultural practices such as the use of fungicides, but how these compounds affect anastomosis formation within and more importantly between networks of the same AM fungal strain remains poorly unexplored. Here, the impact of azoxystrobin, pencycuron, flutolanil, and fenpropimorph at 0.02 and 2 mg L^−1^ were tested in vitro on the anastomosis formation within and between networks of *Rhizophagus irregularis* MUCL 41833. Azoxystrobin and fenpropimorph had a particularly detrimental impact, at the highest concentration (2 mg L^−1^), on the number of anastomoses within and between networks, and for fenpropimorph in particular at both concentrations (0.02 and 2 mg L^−1^) on the number of anastomoses per length of hyphae. Curiously fenpropimorph at 0.02 mg L^−1^ significantly stimulated spore production, while with azoxystrobin, the reverse was observed at 2 mg L^−1^. The two other fungicides, pencycuron and flutolanil, had no detrimental effects on spore production or anastomosis formation within and between networks. These results suggest that fungicides with different modes of action and concentrations differentially affect anastomosis possibly by altering the hyphal tips of AM fungi and may thus affect the capacity of AM fungi to develop large hyphal networks exploring and exploiting the soil at the service of plants.

## Introduction

*Rhizophagus irregularis* forms large mycelial networks via anastomosis between different branches of the same or different hyphae (de la Providencia et al. 2005; Kirk et al. 2008), ultimately connecting plants (Bücking et al. 2016; Wipf et al. 2019). The mechanism of anastomosis has essential functions in fungal mycelium such as heterokaryon formation, intra-hyphal communication, and homeostasis maintenance (Glass et al. 2000). In arbuscular mycorrhizal (AM) fungi, it allows mycelial growth and physical exploration of the environment forming a three-dimensional network with protoplasmic/cytoplasmic flow, genetic material exchange, and exchange of mineral nutrients (Giovannetti et al. 2001, 2015; Mikkelsen et al. 2008; Croll et al. 2009; Daubois et al. 2016; de Novais et al. 2017; Sbrana et al. 2018). This capacity to form anastomoses within and between mycelial networks represents an important mechanism evolved by AM fungi to fulfill their functions in nutrients uptake and transport to the plant. For instance, Avio et al. (2006) noticed a positive correlation between anastomosis frequency in *Glomeraceae* members and growth and P nutrition of *Medicago sativa*.

Agricultural practices such as the application of pesticides can affect spore germination and production, and viability of extraradical mycelium (ERM) (Zocco et al. 2008; Campagnac et al. 2009; Calonne et al. 2012) as well as diversity of AM fungi (Ren et al. 2021; Edlinger et al. 2022). Only a few studies (de Novais et al. 2019a; Rodriguez-Morelos et al. 2021), however, have reported the effects of fungicides on anastomosis formation within a mycelial network and no study, to the best of our knowledge, has reported the effects of fungicides on the capacity of AM fungi to form anastomoses between networks of the same isolate.

The objective of this study was to investigate under in vitro culture conditions the impact of four fungicides (azoxystrobin, pencycuron, flutolanil, and fenpropimorph) with contrasting modes of action on the capacity of *R. irregularis* MUCL 41833 to form anastomoses within and between mycelial networks. *Rhizophagus irregularis* MUCL 41833 was selected because it belongs to a family, the Glomeraceae, (i) widely distributed in agricultural soils (Séry et al. 2018; Guzman et al. 2021) thus susceptible to grow in the presence of fungicides, and (ii) able to form anastomoses within (de la Providencia et al. 2007; Purin and Morton 2011; Pepe et al. 2018) and between (Voets et al. 2006; de la Providencia et al. 2013) mycelial networks.

## Materials and methods

### Biological material

*Rhizophagus irregularis* (Błaszk., Wubet, Renker and Buscot) C. Walker and A. Schüßler as “irregulare” MUCL 41833 was supplied by the Glomeromycota in vitro collection (GINCO—http://www.mycorrhiza.be/ginco-bel) and maintained in association with Ri T-DNA transformed carrot (*Daucus carota* L.) roots on 90-mm diameter Petri plates containing 25 mL of Modified Strullu-Romand (MSR) medium (Declerck et al. 1998).

### Fungicide medium preparation

Four fungicides were supplied by Sigma-Aldrich, Inc. (Darmstadt, Germany) as active ingredients: three systemic fungicides, azoxystrobin and flutolanil which disrupt respiration, and fenpropimorph which inhibits sterol biosynthesis in membranes, and one contact fungicide, pencycuron, which affects the cytoskeleton and motor proteins (Fungicide Resistance Action Committee 2022). The active ingredients were tested at two concentrations (i.e., 0.02 mg L^−1^, a low concentration which poorly affected the development of *R. irregularis*, and 2 mg L^−1^, a high concentration which strongly affected the development of *R. irregularis* (Campagnac et al. 2008, 2009; Zocco et al. 2011)) in the MSR medium, following dissolution in acetone at a concentration of 5 mL L^−1^ of MSR medium. Eight fungicide treatments were considered: azoxystrobin (0.02 and 2 mg L^−1^), flutolanil (0.02 and 2 mg L^−1^), pencycuron (0.02 and 2 mg L^−1^), and fenpropimorph (0.02 and 2 mg L^−1^). Two control treatments were also included in the design: MSR medium without fungicides but supplemented with the same concentration of acetone (MSR^acetone^) and MSR medium alone (MSR^control^).

### Experiment design

#### Impact of fungicides on the extraradical mycelium (ERM) networks of *R. irregularis*

**Fig. 1:**
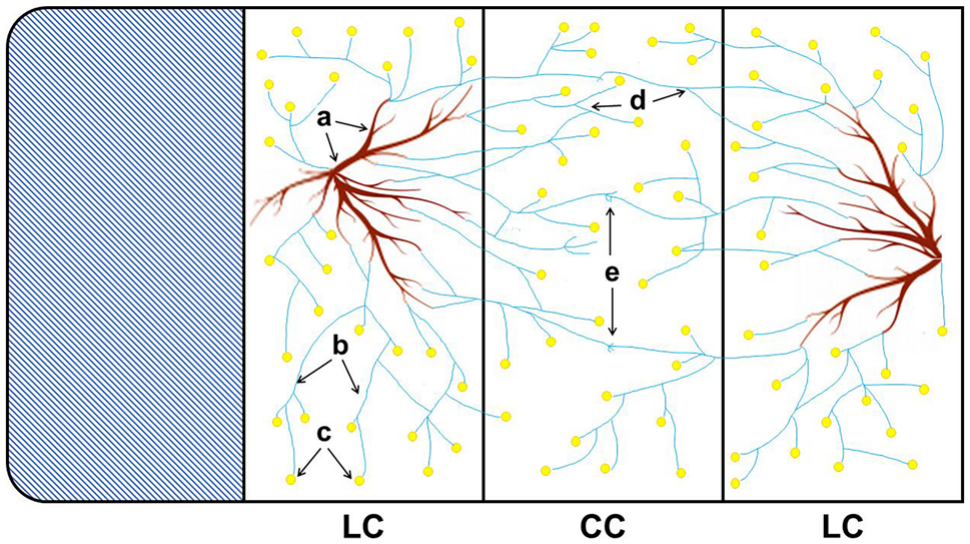
Schematic representation of the quadri-compartmental culture system (whole plate 12.4 × 8.5 cm; each compartment 3.1 × 8.5 cm). A carrot root organ culture (ROC) (**a**) with mycelium (**b**) and spores (**c**) of *R*. *irregularis* MUCL 41833 was placed on the MSR medium in two lateral culture compartments (LC). After 4–6 weeks, the two LCs were covered with a dense extraradical mycelium (ERM) (**b**, **c**). Then, the central compartment (CC) was supplemented with MSR medium without fungicide (MSR^control^) or (MSR^acetone^) or two concentrations (0.02 and 2 mg L^−1^) of azoxystrobin, pencycuron, flutolanil, and fenpropimorph. After a period of 15 days, the CC was covered with ERM. Finally, the anastomosis formation within network (**d**) or between networks (**e**) and ERM growth parameters were assessed in the CC

In vitro cultures of *R*. *irregularis* MUCL 41833 were established in rectangular culture plates comprising 4 adjoining compartments (whole plate 12.4 × 8.5 cm; each compartment 3.1 × 8.5 cm, quadri-PERM, Greiner Bio-One (Kremsmünster Austria)), but only three were used, two lateral compartments (LC) on both sides of one mycelium central compartment (CC) separated by plastic walls (Fig. 1) (Voets et al. 2006).

Thirty milliliters of MSR medium was added to each LC, whereas the CC remained empty. One 1.5 × 1.5 cm sliced piece of gel containing a mycorrhizal root with ERM and approximately 250 spores from a 6-week-old root organ culture (ROC) of carrot was placed on the surface of the MSR medium (Cranenbrouck et al. 2005) in both LCs. The culture plates were incubated in an inverted position at 27 °C in a growth chamber under dark conditions. During the incubation period, the root and mycelium growth was monitored every 48 h. The roots that crossed the partition walls separating the LCs from the CC were trimmed. After 4 to 6 weeks, the two LCs were covered with a dense ERM. The culture plates were selected for their homogeneous development of mycelial network developing in each LC. At that time, the CC was filled with 30 mL of MSR medium without fungicide (MSR^control^) or (MSR^acetone^) or with each of the fungicide treatments (see above), to reach the top of the plastic walls separating the LCs with the CC. After a period of 15 days, the CC was covered with the ERM throughout the depth of the medium. Different parameters were recorded in the CC: (1) the number of newly produced spores, (2) the total hyphal length, and (3) the number of anastomoses within the same network (i.e., originating from the same LC) and between networks (i.e., originating from the two intersecting mycelial networks developing from each LC). In the medium, all anastomoses were numbered, although it was not always possible to verify possible incompatible fusions and non-active anastomoses. The total hyphal length was measured following the method detailed by Tennant (1975) and Declerck et al. (1998). Briefly, gridlines composed of squares of 1 cm^2^ was placed on the bottom of the plates and intersections of hyphae with the gridlines were counted. The length of the mycelium was estimated according to the formula: length (cm) = (number of intersections*area of the compartment (cm^2^)) / (2*length of lines of the grid (cm)). To determine the presence and type of anastomosis, the culture plates were observed under a dissecting microscope Olympus SZ2-ILST, Japan (6.7 to × 40 magnification) and a compound Olympus BH2-RFCA, Japan, with bright-field light illumination (× 40 or × 100 magnification). The experiment was repeated twice with a one-month interval (two experimental repetitions). A total of five replicates that consisted of one culture plate with two LCs and one CC distributed in each of the two experimental repetitions were considered for every treatment.

### Statistical analysis

Levene and Shapiro–Wilk tests were used to confirm homogeneity of variance and normality in distributions of response variables, respectively. Both control treatments (i.e., MSR^acetone^ and MSR^control^) were compared using a one-way ANOVA in order to test the effects of acetone used as fungicide solvent on the AM fungi growth parameters. Because no significant difference was observed between both treatments (result not shown), the impact of fungicides was compared solely to the MSR^acetone^ control. The hyphal length of ERM, number of spores, and the number of anastomoses within the same network and between networks as well as the total number of anastomoses produced per hyphal length in the CC were analyzed with a one-way ANOVA followed by a Tukey HSD post hoc test. Specifically, the *aov* function of the *multcomp* R package (Hothorn et al. 2008) was used, where the experimental repetitions were introduced as random effect. No significant differences were observed between the experimental repetitions. A normal distribution was observed for the hyphal length of ERM, the number of anastomoses between networks and the total number of anastomoses and spores produced per hyphal length. To satisfy the model assumptions (normality and homoscedasticity of the residuals), however, the number of spores produced and the number of anastomoses within the same network were transformed using log10. All data were analyzed with R software version 4.1.2. and statistical significance was established at a 95% confidence level (i.e., when the *P*-value is below 0.05).

## Results

**Fig. 2:**
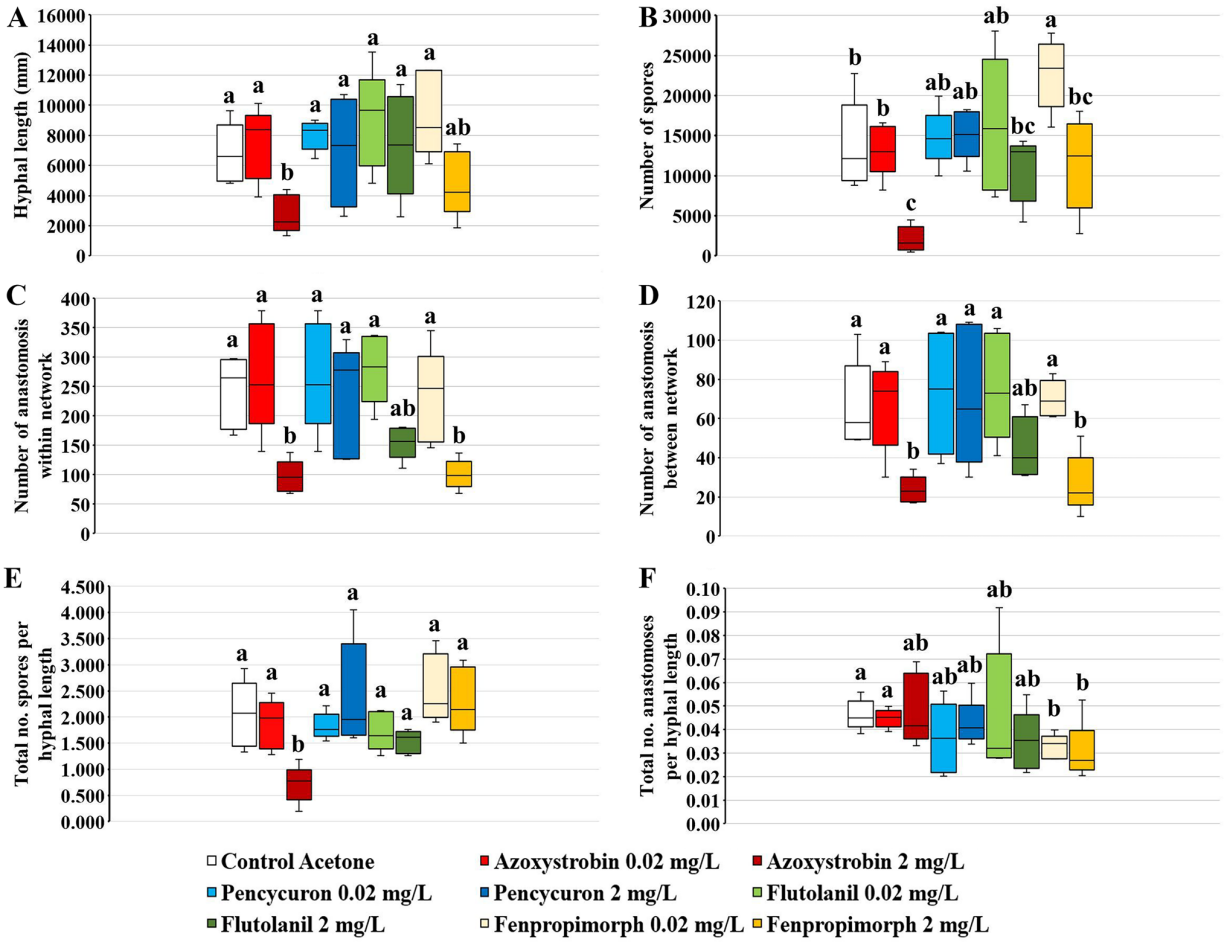
Hyphal length (mm) (**A**), number of spores (**B**) and total number of spores per hyphal length (**E**), hyphal anastomosis within (**C**) and between (**D**) networks and total number of anastomoses per hyphal length (**F**) in extraradical mycelium (ERM) of *Rhizophagus irregularis* MUCL 41833 after 15 days of growth within the central compartment (CC) in the absence (control acetone) or in the presence of two concentrations (0.02 and 2 mg L.^−1^) of azoxystrobin, pencycuron, flutolanil, and fenpropimorph. Horizontal lines within boxes depict means (*N* = 5), boxes depict the interquartile range, and error bars depict ± standard error. For each parameter, the same letters indicate no significant difference between treatments, according to a one-way ANOVA followed by a Tukey HSD post hoc test (*P* ≤ 0.05)

No response variables were affected by pencycuron and flutolanil at either concentration in comparison with the MSR^acetone^ control (Fig. 2A–F).

In contrast, azoxystrobin at the highest concentration (2 mg L^−1^) significantly reduced hyphal length, total number of spores, total number of spores per hyphal length, and total number of anastomoses within the same network or between different networks compared to the MSR^acetone^ control and to azoxystrobin at the lowest concentration (0.02 mg L^−1^) which never differed from the MSR^acetone^ control (Fig. 2A–E). The number of anastomoses per hyphal length was not affected by azoxystrobin, whatever the concentration applied (Fig. 2F). A similar trend was observed in presence of fenpropimorph at the highest concentration (2 mg L^−1^). In presence of fenpropimorph at 2 mg L^−1^, the number of anastomoses within the same network or between different networks were significantly reduced as compared to the MSR^acetone^ treatment and fenpropimorph at the lowest concentration (0.02 mg L^−1^) (Fig. 2C, D). At both concentrations, the total number of anastomoses per hyphal length was significantly reduced by fenpropimorph as compared to the MSR^acetone^ treatment (Fig. 2F). In addition, fenpropimorph at 0.02 mg L^−1^ significantly increased the total number of spores as compared to the MSR^acetone^ and fenpropimorph at 2 mg L^−1^ (Fig. 2B). The hyphal length and total number of spores per hyphal length were not affected by the two concentrations of fenpropimorph (Fig. 2A, E).

## Discussion

The impact of pesticides on the anastomosis of AM fungi has been little studied. For instance, under in vitro conditions, Cardenas-Flores et al. (2011) reported a reduction in the anastomosis of *R*. *clarus* germlings in the presence of 10 mg L^−1^ of fenhexamid and de Novais et al. (2019a) noticed a decrease or null effect in anastomosis on germlings of *Funneliformis mosseae* lineages in growth chamber conditions with fenhexamid at 10 and 20 mg L^−1^ and benomyl at 0.17 and 1.07 mg L^−1^. With chicory plants, de Novais et al. (2019b) noticed also that benomyl at 6.3 mg L^−1^ and fenhexamid at 20 mg L^−1^ decreased the ERM of *F*. *mosseae* but did not affect the anastomosis process.

In our study, differences were observed between active ingredients and concentrations of fungicides. Azoxystrobin and fenpropimorph at 0.02 mg L^−1^ did not affect the total number of anastomoses within and between networks, while at 2 mg L^−1^ a significant decrease was noticed for both parameters. This could be partly related to the decrease in total hyphal length (respectively 63% and 49% compared to the length at 0.02 mg L^−1^) observed with both active ingredients, reducing the probability for hyphae to anastomose. With fenpropimorph, however, the total number of anastomosis per hyphal length at 2 mg L^−1^ as well as 0.02 mg L^−1^ was significantly decreased, which was not the case with azoxystrobin, suggesting a more drastic effect of this active ingredient. These results complement the observations made by Rodriguez-Morelos et al. (2021) on the effects of both active ingredients at 2 mg L^−1^ on the hyphal healing mechanism (HHM) of *R*. *irregularis.* Those authors observed that azoxystrobin and fenpropimorph applied at 2 mg L^−1^ on injured hyphae drastically affected the mechanism of recognition between the cut extremities and therefore wound healing, while no impact was noticed at 0.02 mg L^−1^. They suggested a disruption of the Spitzenkörper, a structure found at the hyphal tip present in many filamentous fungi that mediates hyphal growth direction and hyphal tip morphology by the delivery of secretory vesicles (Riquelme and Sánchez-León 2014). These secretory vesicles have been suggested in AM fungi, although not conclusively demonstrated (de la Providencia et al. 2005). In their study, de la Providencia et al. (2005) and Voets et al. (2006) suggested that fusion-competent hyphae of AM fungi exhibit remote sensing, resulting in growing hyphal tip (GHT) initiation and/or redirection of growth to facilitate contact between participating hyphae. This was clearly shown for the HHM of *Gigaspora* and *Scutellospora* species and suggested in *Glomus*/*Rhizophagus* species (de la Providencia et al. 2005), supporting that the growth of GHTs or hyphae towards each other was due to elicitation of diffusible substance (i.e., secretory vesicles) and that this mechanism was led by one GHT (branch leader) or hyphae in a sequence of signaling-responses. It is not excluded, but must be firmly demonstrated, that in the presence of some fungicides (e.g., azoxystrobin and fenpropimorph), these attributes are “partially” lost, disrupting the directional memory, or resulting in growth arrest, thus reducing/annihilating the anastomosis process. That the number of anastomoses per hyphal length was affected by fenpropimorph at both concentrations, while this was not the case for azoxystrobin, tends to suggest that fenpropimorph was more detrimental to the signaling-response mechanisms and thus to anastomosis formation than azoxystrobin.

Our results with fenpropimorph further support studies conducted under in vitro culture conditions by Campagnac et al. (2008, 2009, 2010) and Zocco et al. (2008, 2011). Those authors demonstrated that this sterol biosynthesis inhibitor at doses equal or above 0.2 mg L^−1^ decreases ERM growth of *R*. *irregularis*. It is well known that fenpropimorph inhibits ergosterol biosynthesis (Debieu et al. 2000), which is the typical sterol in the vast majority of fungi but absent from AM fungi, while 24-methyl and 24-ethyl cholesterol are the two major sterol compounds in AM fungi (Fontaine et al. 2001; Olsson et al. 2003). Interestingly, Steinberg (2007) suggested that sterol-rich membrane domains, termed lipid rafts, might support hyphal tip growth by facilitating apical endocytosis and providing an apical scaffold that organizes the cytoskeleton. Therefore, the impact of fenpropimorph on anastomosis possibly might be attributable to its impact on secretory vesicles as mentioned above and/or to the inhibition of specific sterols in hyphal tips. Curiously, a stimulatory effect on spore production was observed at the lowest dose of fenpropimorph compared to the control. It is difficult to speculate what mechanism might be involved but may indicate a stress response to fungicide exposure at small doses.

Azoxystrobin exerts its fungicidal action by blocking electron transport in the mitochondrial respiratory chain in fungi (Balba 2007). This drastically reduces energy production, thereby inhibiting growth of the fungus as was shown by the 63% decrease in total hyphal length and 84% reduction in spore production between the 0.02 and 2 mg L^−1^ concentrations in our experiment. This also resulted in a significant decrease in the total number of anastomosis within and between networks, while expressed in the number of anastomoses per hyphal length, no impact was detected. This suggested that this active ingredient has probably less impact than fenpropimorph on the remote sensing mechanism between hyphae.

Neither pencycuron (a phenylurea highly specific to sensitive strains of *Rhizoctonia solani* – Young et al. 2012) nor flutolanil (a highly systemic phenyl-benzamide used against Basidiomycota pathogens – Zhao et al. 2019) affected mycelium growth and anastomosis of *R*. *irregularis* at either concentration. This corroborates the study of Rodriguez-Morelos et al. (2021) showing an absence of effects of these fungicides at the same concentrations on the HHM of *R*. *irregularis* and the work of Buysens et al. (2015), which noticed that flutolanil at 0.1 mg L^−1^, did not affect ERM grown. Thus, the absence of impact suggests that the enzymes involved in cell division in hyphal tips or the respiration pathway in *R*. *irregularis* are different or less sensitive than those in *R*. *solani* or Basidiomycota pathogens, respectively.

In conclusion, this work reports for the first time the direct effects of fungicides on hyphal anastomosis between different networks of the same strain of *R. irregularis*, complementing results already obtained on anastomoses between hyphae within the same mycelial network. The effects vary with active ingredient and concentration, with the broad-spectrum fungicides azoxystrobin and fenpropimorph being the most detrimental at 2 mg L^−1^. This concentration often is considered above field dosage for azoxystrobin. For example, Buysens et al. (2015) suggested that 0.75 mg L^−1^ concentration in vitro corresponds to 1500 g ha^−1^ (formulation Amistar) to control *R*. *solani* in potato. Zocco et al. (2011) suggested that fenpropimorph at 0.02 and 2 mg L^−1^ corresponds to 0.017 and 0.17 times, respectively, the recommended values for field application (formulation Corbel). Therefore, even if fenpropimorph appears more detrimental than azoxystrobin at the same concentration, its effects may be less marked in the field because of the lower dose of application. It is important to note that the impact of fungicides on AM fungi under in vitro culture conditions can only partially reflect field situations, where edaphic factors (e.g., soil physico-chemistry and microbial populations) determine fungicide degradation. In vitro culture, however, allows non-destructive investigation of the role of fungicides on AM fungi under conditions exempt of confounding effects. Therefore, both approaches (within field and in vitro) have complementary strengths to investigate and determine the role of fungicides on the non-target AM fungi. Our results support the hypothesis that fungicides may negatively affect anastomosis by altering the hyphal tips of AM fungi and may thus affect the ability of AM fungi to develop large networks of hyphae exploring and exploiting the soil to serve plants.

## Data Availability

The datasets generated during the current study are available from the corresponding author on reasonable request.
